# Vaccines and Drug Therapeutics to Lock Down Novel Coronavirus Disease 2019 (COVID-19): A Systematic Review of Clinical Trials

**DOI:** 10.7759/cureus.8342

**Published:** 2020-05-28

**Authors:** Akshaya S Bhagavathula, Wafa A Aldhaleei, Alessandro Rovetta, Jamal Rahmani

**Affiliations:** 1 Public Health, Institute of Public Health, College of Medicine and Health Sciences, United Arab Emirates University, Al Ain, ARE; 2 Gastroenterology, Sheikh Shakhbout Medical City, Abu Dhabi, ARE; 3 Mathematical, Statistical and Epidemiological Models, Research and Disclosure Division, Mensana SRLS, Brescia, ITA; 4 Community Nutrition, National Nutrition and Food Technology Research Institute, Shahid Beheshti University of Medical Sciences, Tehran, IRN

**Keywords:** 2019 novel coronavirus, covid 19, treatment choices, vaccines, drug therapeutics, sars-cov-2 (severe acute respiratory syndrome coronavirus -2)

## Abstract

The ongoing novel coronavirus disease 2019 (COVID-19) pandemic has been responsible for millions of infections and hundreds of thousands of deaths. To date, there is no approved targeted treatment, and many investigational therapeutic agents and vaccine candidates are being considered for the treatment of COVID-19. To extract and summarize information on potential vaccines and therapeutic agents against COVID-19 at different stages of clinical trials from January to March 2020, we reviewed major clinical trial databases such as ClinicalTrials.gov, WHO International Clinical Trials Registry Platform (ICTRP), and other primary registries between January and March 15, 2020. Interventional studies at different phases under the COVID-19 pipeline were included. A total of 249 clinical trials were identified between January to March 15, 2020. After filtering observational studies (194 studies), a total of 56 interventional trials were considered. The majority of clinical trials have been conducted on chloroquine (n=10) and traditional Chinese medications (TCMs; n=10), followed by antivirals (n=8), anti-inflammatory/immunosuppressants (n=9), cellular therapies (n=4), combinations of different antivirals therapies (n=3), antibacterial (n=1), and other therapies (n=5). Five vaccines are under phase I, and there are a couple of phase III trials on the Bacillus Calmette-Guérin (BCG) vaccine under investigation among healthcare workers. Many novel compounds and vaccines against COVID-19 are currently under investigation. Some candidates have been tested for other viral infections and are listed for clinical trials against the COVID-19 pipeline. Currently, there are no effective specific antivirals or drug combinations available for the treatment of COVID-19.

## Introduction and background

In early 2020, an outbreak of atypical pneumonia in Wuhan, China caused by a novel coronavirus [Severe acute respiratory syndrome coronavirus 2 (SARS-CoV-2)] emerged as a global pandemic [1]. The World Health Organization (WHO) initially named it 2019-nCoV and later officially termed it novel coronavirus disease 2019 (COVID-19) [2]. SARS-CoV-2 is the seventh member of the coronavirus family to infect humans and has symptoms ranging from fever, cough, myalgia, fatigue, and pneumonia to those indicating fatal severe acute respiratory distress syndrome (SARS) [3,4].

COVID-19 is a potential zoonotic disease that may have originated from bats [5]. The study by** **Chan et al. demonstrated the process of human-to-human transmission of COVID-19, but the estimated reproduction number, R0, was not consistent; however, using early information, the WHO estimated R0 to be 1.4-2.5 [6,7]. The SARS-CoV-2 viral load can be found in the angiotensin-converting enzyme 2 (ACE2) cells reported in the deep respiratory tract samples of humans. In most cases, COVID-19 is a self-limited infection and presents mild or no symptoms in the early incubation phase [8]. However, infected asymptomatic patients can transmit the disease during the incubation period, which has caused great difficulty in controlling the COVID-19 pandemic. Specifically, the elderly population and individuals with underlying chronic diseases such as hypertension, cardiovascular disease, diabetes, and chronic obstructive pulmonary disease are highly susceptible to SARS-CoV-2, and infection can lead to adverse outcomes, such as acute respiratory distress syndrome (ARDS) and cytokine storm [1,3,6,9]. Because there is currently no vaccine and therapy to treat COVID-19, this contagious disease has recorded at least five million cases and more than 320,000 deaths across the globe. In this review, we summarize various potential vaccines and therapeutic agents at different stages of clinical investigation for COVID-19 from January to March 2020.

## Review

1. Methods

In this review, we provide a brief overview of the portfolio of potential vaccines and therapeutic agents for COVID-19 at various phases of clinical trials. Because many of these candidates are under the early stages of investigation and have not entered the peer-reviewed literature, we reviewed the websites of major clinical trial databases including ClinicalTrials.gov (CT.gov), WHO International Clinical Trials Registry Platform (ICTRP), and other primary registries to identify information available between January and March 15, 2020 (Table 1) [10.11].

Two of the authors (WAA and JR) checked all potential candidates under the COVID-19 pipeline. The information retrieved from the clinical trial registries includes therapeutic agents, clinical phases, types of intervention, age group, gender, inclusion and exclusion criteria, and outcome measures. Only interventional trials were considered, and detailed characteristics of these clinical trials have been provided. We assessed only the English version of the included registries. We excluded registries that were non-interventional, and those at pre-clinical stages. Information was summarized based on the information provided on each of the clinical trial websites.

2. Results

A total of 249 clinical trials were identified between January and March 15, 2020. After filtering out observational trials (194 studies), a total of 56 interventional trials were considered. A summary of major COVID-19 treatment candidates under various phases of clinical development are summarized in Table 2.

Thirty-two trials were registered in the Chinese clinical trials registry and the remaining 23 in US ClinicalTrials.gov. Many trials are under investigation at phase IV (n=31) while others are at phase III (n=8), phase II (n=9), and phase I (n=8). The majority of the clinical trials are being conducted on chloroquine (n=10) and traditional Chinese medications (TCMs; n=10), followed by antivirals (n=8), anti-inflammatory/immunosuppressants (n=9), cellular therapies (n=4), combinations of different antivirals therapies (n=3), antibacterial (n=1), and other therapies (n=5). Interestingly, the safety profile of five vaccines are under phase I investigation in healthy volunteers, and a couple of phase III trials are investigating the efficacy of the Bacillus Calmette-Guérin (BCG) vaccine among healthcare workers (Table 3). An overview of these vaccines is presented below.

2.1. Vaccines

2.1.1. Artificial antigen-presenting cells (aAPC) vaccine: acellular aAPCs are promising immunotherapeutic agents that can efficiently stimulate and amplify antigen-specific CD4+ T cells [12]. ACE2 receptors mediate the binding of the SARS-CoV-2 spike protein for viral replication. The primary aim of the aAPC vaccine is to use genetically modified aAPC to immune reactivate the T cells, thus treating and preventing COVID-19. Currently, the aAPC vaccine for COVID-19 treatment and prevention is at phase I and is recruiting healthy volunteers from the age of six months to 80 years in China (NCT04299724). This research is carried out by scientists from Shenzhen Geno-Immune Medical Institute and is expected to investigate the safety and immunity reactivity of the COVID/aAPC vaccine. This study is expected to be completed in 2023 and 2024.

2.1.2. ChAdOx1 vaccine: the ChAdOx1 nCoV-19 vaccine is an adenovirus vaccine vector developed by the Clinical Biomanufacturing Facility at the University of Oxford, UK. The ChAdOx1 nCoV-19 vaccine aims to develop a strong immune response from a single dose and inhibit the replication of the virus. The ChAdOx1 nCoV-19 vaccine contains the genetic sequence of the COVID-19 surface spike protein, causing the immune system to attack the SARS-CoV-2 virus [13]. Currently, ChAdOx1 is in phase I/II trial and is expected to enroll at least 1090 healthy subjects between 18 to 55 years of age from the UK in a randomized sequential manner (NCT04324606); results are expected by mid-2021.

2.1.3. Lentiviral (LV-SMENP-DC) vaccine and antigen-specific cytotoxic T lymphocytes (CTLs): the LV-SMENP is an innovative approach using COVID-19 minigenes engineered into a vaccine and was developed by the Shenzhen Geno-Immune Medical Institute, China. LV-SMENP is developed from multiple genes through the lentiviral vector system (NHP/TYF) to express COVID-19 antigens, causing dendritic cell (DC) modifications and T cell activation. CTLs are activated by the LV-DC present in the COVID-19-specific antigen. After LV-DC vaccination, antigen-specific CTLs are prepared in 7-21 days and administered to the subjects through subcutaneous injection or intravenous infusion [14]. LV-SMENP is currently undergoing phase I/II multicenter trial in healthy volunteers and COVID-19-infected patients among children (>6 months), adults and older population (≤80 years) to evaluate its safety. The study is at the recruitment phase and the results are expected by 2024 (NCT04276896).

2.1.4. mRNA-1273 vaccine: mRNA-1273 is a novel lipid nanoparticle (LNP)-encapsulated mRNA-based vaccine that encodes a full-length, prefusion stabilized spike protein from SARS-CoV-2 virus. The mRNA-1273 vaccine was developed by the National Institute of Allergy and Infectious Diseases (NIAID), USA, and is currently in phase I of clinical development to assess the safety, reactogenicity, and immunogenicity of the vaccine in healthy volunteers (NCT04283461). The current recruitment is ongoing at Emory Vaccine Center in Georgia, National Institute of Health, Maryland, and Kaiser Permanente Washington Health Research Institute, Washington, USA. This open-label, multiple-arm interventional trial is expected to enroll 105 participants who are 18-99 years of age and is expected to be completed in 2021.

2.1.5. Ad5-nCoV: Ad5-nCoV is the first novel genetically engineered vaccine for COVID-19 developed by CanSino Biologics and the Beijing Institute of Biotechnology, China. The vaccine utilizes replication-defective adenovirus type 5 as a vector to express the SARS-CoV-2 spike protein. Currently, the AD5-nCoV vaccine is in active phase I clinical trial to investigate its safety, reactogenicity, and immunogenicity in healthy volunteers between 18-60 years of age (NCT04313127). The study is estimated to enroll 108 participants from Wuhan who are COVID-19-negative in the screening tests and is expected to be completed in 2022.

2.1.6. BCG vaccine: since 1921, the BCG vaccine has been widely used to prevent tuberculosis and leprosy [15]. However, there is currently no evidence that the BCG vaccine is effective against coronavirus infections. Researchers from Australia and UMC Utrecht and Radboud University, Netherlands believe that the BCG vaccine can help to bolster the immune system, thereby reducing the infection rates of SARS-CoV-2. A couple of phase III trials are ongoing to evaluate the efficacy of the BCG vaccine in reducing the incidence of COVID-19 at various children hospitals in Western Australia (NCT04327206). Researchers from UMC Utrecht and Radboud University, Netherlands are investigating the efficacy of BCG vaccination in reducing absenteeism among healthcare workers involved in COVID-19 patient care (NCT04328441). The results of these two studies are expected by the end of 2020.

2.2. Cellular Therapies

2.2.1. Mesenchymal stem cells (MSCs): MSCs are widely used cell-based therapies with superior efficacy in immune-mediated inflammatory diseases such as systemic lupus erythematosus (SLE) and graft-versus-host disease (GVHD) [16]. The direct interaction of MSCs with immune cells activates the toll-like receptor (TLR) to simulate pathogen-associated molecules such as lipopolysaccharide (LPS) or double-stranded RNA from a virus such as COVID-19 [17]. A recent study assessed the immunomodulating efficacy of MSCs in seven COVID-19 patients for 14 days after administration. Within two days of MSC transplantation, patients demonstrated a significant decrease in the C-reactive protein, reactivation of cytokinin-secreting immune cells, and a decrease in TNF-α cells [18]. Currently, two trials in phase I (ChiCTR2000030300) and phase II (NCT04269525) are under investigation for critically ill COVID-19 patients with pneumonia. Since the phase II trial is planned only for 10 COVID-19 patients in critical condition, results are expected by the end of September 2020. The study is sponsored by Tuohua Biological Technology Ltd and in collaboration with Zhongnan Hospital of Wuhan University, China.

2.2.2. Convalescent plasma: to provide immediate immunity to susceptible persons, passive antibodies are administered to prevent or treat an infection. Recent focus has been on human convalescent serum during the viral epidemic for its use in the prevention and treatment of COVID-19 [19]. Two trials are gearing up to investigate the efficacy (NCT04332380) and safety (NCT04333355) of convalescent plasma against COVID-19. The phase II efficacy trial is conducted by the researchers from the Universidad del Rosario, Colombia, and the phase I safety trial is sponsored by Hospital San Jose Technologico de Monterrey, Mexico. Both trials are expected to complete by the end of 2020. Astonishingly, there are 47 ongoing studies registered to evaluate the efficacy of convalescent plasma in the COVID-19 patients, of which 22 are randomized controlled trials (RCTs).

2.3. Anti-Inflammatory/Anti-Allergic Agents and Immunosuppressants

The COVID-19 infection is characterized by an overexuberant inflammatory response that worsens the symptoms. Researchers have identified that the SARS-CoV-2 virus increases the expression of multiple proinflammatory cytokines (TNF-α, IFN-γ, IL-6, and MCP1) in the serum, indicating that the inflammatory storm may be involved in the progression of COVID-19 [20]. Thus, the host inflammatory response to COVID-19 is an important factor leading to lung damage and subsequent mortality. Therefore, anti-inflammatory/anti-allergic and immunosuppressant drugs, alone or in combination, are crucial COVID-19 therapies aiming to limit the excessive inflammatory response to counter the infection. Several potential drugs including meplazumab, fingolimod, bevacizumab, baricitinib, tranilast, Tozumab+adamumab, cerrokin (recombinant human interferon-alpha 1beta), and steroidal therapies are under investigation against SARS-CoV-2.

2.3.1. Meplazumab: CD147 is present in active inflammatory cells and by binding with cyclophilin A (CyPA), CD147 participate in the regulation of cytokine secretion and leukocytes chemotaxis [21]. Meplazumab acts as an anti-CD147 humanized monoclonal antibody that blocks virus invasion and attenuating inflammation [22]. Currently, it is undergoing a phase I/II trial at the Tang-Du hospital, China (NCT04275245) to assess the therapeutic effect with 28 days of administration of human meplazumab injection. The results of this ongoing trial are expected by the end of 2020.

2.3.2. Fingolimod: fingolimod is a sphingosine 1-phosphate (S1P) receptor modulator that is used for immune therapy in patients with multiple sclerosis [23]. Prior studies have described the use of immune modulators together with ventilator support in patients with severe pulmonary edema to prevent the development of ARDS [24,25]. A phase II study (NCT04280588) at Fujian Medical University, China aims to determine the efficacy of fingolimod for treating severe pneumonia in COVID-19 patients. The results of this ongoing interventional trial are expected by July 2020.

2.3.3. Bevacizumab: bevacizumab is a humanized monoclonal anti-vascular endothelial growth factor (VEGF) antibody. VEGF receptors are present on endothelial cells that produce various inflammatory and epithelial cells acting as potential vascular permeability inducer. Prior studies have identified increased levels of VEGF in patients with ARDS [26,27]. Given the causal link between ARDS and increased pulmonary edema and vascular permeability, the researchers believed that bevacizumab might be a promising therapeutic agent in severe cases of COVID-19. Bevacizumab may reduce the levels of VEGF caused by hypoxia, severe inflammation, and upregulation of the infected tract epithelium; therefore, it can suppress the edema in patients with COVID-19. Currently, a phase II/III, open-label, pilot study on 20 patients is ongoing (NCT04275414) to assess the efficacy and safety of bevacizumab for the treatment of severe or critical patients with COVID-19 pneumonia (BEST-CP study). The results are expected by the end of May 2020.

2.3.4. Baricitinib: baricitinib is a highly selective Janus kinase (JAK) inhibitors approved for the treatment of rheumatoid arthritis and is also found to have an antiviral effect [28]. The JAK-dependent pathways are involved in producing a variety of cytokines and increased levels of inflammatory factors including IL-6 and IL-7 that can involve in the pathogenesis of COVID-19 [29]. Therefore, baricitinib might act as a double-edged sword by inhibiting the inflammatory factors and also showing antiviral activity against the SARS-CoV-2 virus. Currently, investors from the hospital of Prato, Italy have registered a phase II/III, open-label, two-week trial to assess the efficacy of baricitinib in combination with antiviral therapy in patients with mild to moderate COVID-19 infection (NCT04320277). The study is expected to recruit 200 patients from mid-May 2020.

2.3.5. Tranilast: tranilast is an analog of a tryptophan metabolite, identified as a potential anti-allergic agent and used for the treatment of inflammatory diseases [30]. By releasing chemical mediator histamines, tranilast inhibits immunoglobulin E (IgE) antibodies from mast cells, and it also showed promising results in controlling bronchial asthma in children [31]. It is a potential cytokine modifier, and also a potent inhibitor of many inflammatory mediators, cytokines, and chemokines. It is proven to have suppressive effects on histamines, COX-2, IL-1β, IL-2, IL-5, IL-6, IL-8, eotaxin-1, and TGF-β1 [30]. Reviewing its benefits, researchers from the University of Science and Technology of China registered a phase IV, interventional trial to evaluate the efficacy and safety of tranilast in treating patients with COVID-19 pneumonia (ChiCTR2000030002).

2.3.6. Tozumab+adamumab: tozumab is an anti-insulin-like growth factor type 1 receptor (IGF-1R) antibody that showed substantial responses in treating a small number of cancer patients with selective tumor types such as Ewing sarcoma and thymoma [32]. However, the exact reason for proposing tozumab in combination with adamumab for the treatment was severe, and critically ill COVID-19 patients with pneumonia is unclear. A phase IV, randomized, parallel controlled trial was registered by the Shanghai General Hospital at ChiCTR (ChiCTR2000030580) in March 2020.

2.4. Antivirals

Antiviral drugs are essential for the prevention and treatment of respiratory illness and are potentially important to halt coronavirus replication. Antiviral drugs used for influenza virus, respiratory syncytial virus (RSV), and other conditions are under investigation for COVID-19 [33]. Currently, there is no specific antiviral drug that effectively treats COVID-19. In this regard, several antivirals are under investigation in phase II and phase III clinical trials. A detailed list of these drugs is presented in Table 3.

Favipiravir is a viral RNA polymerase inhibitor effective against different types of influenza viruses and other RNA viruses such as arenaviruses, bunyaviruses, and filoviruses [34]. Given its broad-spectrum activities, favipiravir is considered a potentially promising drug for treating COVID-19 and is currently in phase II clinical trial (ChiCTR2000029996) to investigate its efficacy in treating patients with COVID-19 pneumonia. Antiretroviral drugs such as Prezcobix® (darunavir/cobicistat; Janssen Pharmaceutica, Beerse, Belgium) and lopinavir/ritonavir are known to have superior safety profiles in HIV/AIDS patients and are currently undergoing phase III clinical trials for the treatment of COVID-19 (NCT04321174). Remdesivir, a potential viral RNA polymerase inhibitor, is an investigational medicine for the Ebola virus with undetermined safety and efficacy [35]. Due to the urgency of the COVID-19 pandemic, multiple phase III clinical trials for remdesivir are underway (NCT04252664). The characteristics of these antiviral are described in Table 3.

Along with these antivirals, the antimalarial drugs hydroxychloroquine and chloroquine have also demonstrated versatile antiviral activity against RNA viruses such as poliovirus, influenza A and B virus, hepatitis A virus, hepatitis C virus, influenza A H5N1 virus, Ebola virus, and Zika virus [36]. Researchers from France have demonstrated the benefits of chloroquine in a small group of COVID-19 patients whereas an RCT from China showed no difference in the recovery rates in patients with mild to moderate COVID-19 [37,38]. There are currently many clinical trials underway attempting to generate robust evidence regarding the efficacy and safety of chloroquine and hydroxychloroquine in the treatment of COVID-19. In addition to these medications, several TCMs and other miscellaneous agents are under investigation for some possible effects against COVID-19 (Table 3).

We also reviewed the information of recent clinical trials for COVID-19 that have not yet been initiated, which are summarized in Table 4. There are currently limited treatment options available for COVID-19, and it will take several months to years to investigate potential treatments for their efficacy and safety. In the meantime, several researchers and public health agencies are repurposing medications with decent efficacy for other similar diseases. For instance, thalidomide, an immunomodulatory and anti-inflammatory agent, is known for its effects in stimulating T cells, inhibiting cell proliferation and anti-inflammation, and reducing lung injury and pulmonary fibrosis [39]. A couple of phase II trials have registered thalidomide either alone (NCT04273529) or in combination with adjuvant therapies (NCT04273581) in severe COVID-19 patients.

From China, the SARS-CoV-2 has spread around the world to emerge as an ongoing global pandemic [1,4,5]. Despite tremendous global efforts to reduce the spreading of SARS-CoV-2, human-to-human transmission continues apace leading to high morbidity and mortality. Most mortalities occur in individuals of older age and those with underlying chronic diseases such as hypertension, diabetes, and cardiovascular disease, which have compromised their immune system [40]. During the past months, there has been some significant progress in the development of therapeutics and vaccines for the treatment of COVID-19. However, these are still in the early stages. Through this review, we extracted and summarized the information regarding vaccines and therapeutics at different stages of clinical trials. We carried out this review by comprehensively searching the websites of major clinical trial databases to identify potential therapeutic agents and vaccines for COVID-19.

Several vaccines are currently under development and some have begun to be tested on healthy volunteers. Most vaccine candidates are developed from recombinant DNA and mRNA, or inactivated whole-virus (IWV) [41]. Given the close genetic relationship between SARS-CoV and SARS-CoV-2, the current SARS-CoV-2 vaccines may provide potential cross-protective effects and provide better efficacy to prevent COVID-19 [42].

Two antimalarial drugs, chloroquine and hydroxychloroquine, together with azithromycin, have been reported to be ineffective for the treatment of COVID-19 [37,43]. However, there was no clinical evidence of viral clearance after seven days of treatment [38]. Considering the seriousness of COVID-19 pandemic, several novel therapeutic agents and some repurposed agents are under investigation in phase II and III clinical trials to test their efficacy against COVID-19. The major challenge in drug discovery and development is to demonstrate that the medicine is effective and safe in both small experimental setups and in human clinical settings.

Apart from supportive care, such as extracorporeal membrane oxygenation for severe COVID-19 cases and oxygen inhalation in mild cases, passive antibody therapy and cell-based therapies have clearly demonstrated efficacy and safety in many clinical trials [44]. Convalescent plasma or immunoglobulins are used as a last resort to improve the survival of COVID-19 patients in deteriorating conditions [45]. The use of convalescent plasma collected from patients recovered from infection has resulted in a short hospital stay and lower mortality rate in treated patients [46]. In 2014, the WHO recommended the use of convalescent plasma as empirical treatment in the Ebola outbreak [47]. Convalescent plasma obtained from recovered patients may provide antibodies capable of suppressing the viremia. However, more research is needed to confirm its efficacy in COVID-19 patients. In general, viremia peaks during the first week of infection and causes a primary immune response within 10-14 days. Thus, investigations focusing on the early transfusion of convalescent plasma and its effect on viral suppression are warranted.

In addition to the development of vaccines and therapeutics, numerous TCMs are also under investigation for treating COVID-19. The novel discovery of artemisinin from sweet wormwood (Artemisia annua) has made a significant difference in the management of malaria treatment globally [48]. TCMs are mainly investigated in critically ill patients. Fuzheng Huayu (FZHY) preparations are known to have an antifibrotic effect and have been approved by USFDA for phase II trials in patients with chronic hepatitis C [49]. Currently, the FZHY formula of six traditional herbs (radix Salvia miltiorrhiza, pollen pini, Semen Persicae, Gynostemma pentaphyllum, Cordyceps, and Fructus Schisandrae Chinensis) is undergoing phase II trials for the treatment of pulmonary fibrosis in COVID-19 patients.

3. Limitations

This review has some limitations to consider. First, this systematic review was carried out on the basis of protocols registered in the various clinical trial websites. Secondly, despite the fact that the search strategy used in this review was limited to until March 30, 2020, we may have missed some of the important trials that were registered in the later stage of this period. Third, due to the pressing nature of the active COVID-19 pandemic, which resulted in a rapidly evolving research scene, many studies that are currently underway have not been included. Lastly, we did not perform the risk of bias to assess the quality of these clinical trial registries.

## Conclusions

In this review, we summarized the different approaches currently under investigation to provide effective treatment and thereby reduce the incidence and mortality related to COVID-19. Looking ahead, the most feasible options meriting further evaluation in clinical trials for the ongoing COVID-19 pandemic include monotherapy or combinations of antivirals, monoclonal antibodies with anti-inflammatory therapies, or immune suppressants that have a protective effect. In the long term, effective vaccines are essential to break the chain of transmission from infected human/animal to susceptible hosts and thus control the spread of SARS-CoV-2.

## Appendices

**Table 1:** List of websites searched for identifying the potential therapeutic agents for COVID-19 COVID-19: coronavirus disease 2019

Country/region	Organization/resource	Website
USA	ClinicalTrials.gov (a service of the US National Institute of Health)	http://www.clinicaltrials.gov/
China	Chinese Clinical Trials Registry (ChiCTR)	http:// http://www.chictr.org.cn/
Japan	Japan Primary Registries Network (JPRN)	http://rctportal.niph.go.jp/
South Korea	Clinical Research Information Service (ICRiS)	http://www.ncrc.cdc.go.kr
Australia and New Zealand	Australia New Zealand Clinical Trials Registry (ANZCTR)	http://www.anzctr.org.au/
European Union	EU Clinical Trials Registry (EU-CTR)	http://www.clinicaltrialsregister.eu/
United Kingdom	International Standard Randomised Controlled Trial Number Register (ISRCTN)	http://www.isrctn.org/
Germany	German Clinical Trials Register (DRKS)	http://drks.neu.uniklinik-freiburg.de
Netherlands	Netherlands National Trial Register (NTR)	http://www.trialregister.nl
Iran	Iranian Registry of Clinical Trials (IRCT)	http://www.irct.ir

**Table 2:** Progression of the clinical development of new anti-COVID-19 candidates from January to March 2020 COVID-19: coronavirus disease 2019; aAPC: artificial antigen-presenting cells; LV: lentiviral; DC: dendritic cell; CTLs: cytotoxic T lymphocytes; UC-MSCs: umbilical cord-derived mesenchymal stem cells; BCG: Bacillus Calmette-Guérin

Phase I	Phase II	Phase III	Phase IV
aAPC vaccine; ChAdOx1 vaccine; convalescent plasma; LV-SMENP-DC and antigen-specific CTLs; meplazumab; mRNA-1273 vaccine; recombinant novel coronavirus vaccine (adenovirus type 5 vector); stem cell therapy	Bevacizumab; chloroquine phosphate; favipiravir; Fuzheng Huayu tablet; fingolimod; hydrochloriqine+azithromycin+tocilizumab; methylprednisolone; plasma; UC-MSCs	BCG vaccine; baricitinib; darunavir+cobicistat; hydroxychloroquine; lopinavir/ritonavir; remdesivir; Triazavirin	Alpha-lipoic acid; arbidol tablets; Ba-Bao-Dan; carrimycin; Cerrokin; (recombinant human interferon-alpha 1beta); chloroquine phosphate; corticosteroid intervention; ebastine+interferon-alpha aerosol inhalation+lopinavir; ganovo+ritonavir+/-Interferon; honeysuckle oral liquid; hydroxychloroquine sulfate; Inhalate the mycobacterium vaccae; Jinye Baidu granule; Kangbingdu granules; Kesuting syrup; Lianhua Qingwen capsules; Novaferon atomization inhalation+lopinavir/ritonavir tablets; polyinosinic-polycytidylic acid Injection; Shenqi Fuzheng injection; sodium aescinate injection; tozumab and adamumab; tranilast; Xiyanping injection

**Table 3:** Characteristics of the ongoing clinical trial performed on COVID-19 patients included in the review *Description of the numbers under the header Exclusion: 1 - pregnant or lactating women; 2 - allergic/hypersensitivity/contraindication to the drug; 3 - participating in other clinical trials; 4 - COVID-19 not confirmed; 5 - uncooperative/unable to provide consent/mental illness; 6 - severe comorbidities/severe illness/shock/organ dysfunction; 7 - confirmed bacterial or fungal infections COVID-19: coronavirus disease 2019; aAPC: artificial antigen-presenting cells; DC: dendritic cell; BCG: Bacillus Calmette-Guérin; UC-MSCs: umbilical cord-derived mesenchymal stem cells; RCT: randomized controlled trial; BMI: body mass index; HIV: human immunodeficiency virus; CT: computed tomography; RT-PCR: reverse transcription-polymerase chain reaction; SARS CoV-2: severe acute respiratory syndrome coronavirus 2; CRP: C-reactive protein; ESR: erythrocyte sedimentation rate; TNF: tumor necrosis factor; RICU: respiratory intensive care unit; FVC: forced vital capacity

Agent	Trial phase	Intervention	Age	Gender	Inclusion	Outcome measures	Exclusion*	Reference
Vaccines								
aAPC vaccine	1	Single-arm	6 months–80 years	Both	COVID-19-positive volunteers and signed an informed consent	Frequency of vaccine events and 28-day mortality	1, 2, 3	NCT04299724
mRNA-1273 vaccine	1	Sequential	18–55 years	Both	Signed informed consent and BMI of 18-35	Frequency of any serious adverse events (SAEs)	1, 2, 3	NCT04283461
Recombinant novel coronavirus vaccine (adenovirus type 5 vector)	1	Sequential	18–60 years	Both	HIV-negative test and normal lung CT	Safety indexes of adverse reactions	1, 2, 3	NCT04313127
ChAdOx1 vaccine	1/2	Single-arm	18–55 years	Both	Signed informed consent and an agreement to refrain from blood donation during the study course	Assess the efficacy and safety of the candidate ChAdOx1 nCoV-19	1, 2, 3	NCT04324606
Lentiviral minigene vaccine (LV-SMENP-DC)	1/2	Single-arm	6 months–80 years	Both	RT-PCR confirmed COVID-19	Clinical improvement based on the 7-point scale; lower Murray lung injury score	1, 2, 3	NCT04276896
BCG vaccine	3	Parallel	>18 years	Both	Hospital personnel taking care of patients with SARS CoV-2 infection	Healthcare workers' absenteeism. COVID-19 incidence by 12 months	1, 2, 3	NCT04328441 NCT04327206
Cellular therapy								
Mesenchyme stem cells (MSCs)	1	Non-RCT	18–75 years	Both	Pneumonia severity index (PSI) III - V level, or arterial blood oxygen partial pressure (PaO2/oxygen concentration (FiO2) (P/F) 300 mmHg) or less	Time to clinical recovery; exacerbation (transfer to RICU) time	1, ,5, 6	ChiCTR2000030300
Convalescent plasma	1	Single-arm	>18 years	Both	RT-PCR confirmed COVID-19	Side effects	2, 3, 6	NCT04333355
UC-MSCs	2	Single-arm	18–75 years	Both	Critical COVID-19 pneumonia with ICU stays of <48 hours	Oxygenation index	NA	NCT04269525
Convalescent plasma	2	Single-arm	18–60 years	Both	RT-PCR confirmed COVID-19	Change in viral load	1, 2, 6	NCT04332380
Anti-inflammatory/anti-allergic agents and immunosuppressants								
Meplazumab	1/2	Single-arm	18–75 years	Both	New COVID-19 patients with pneumonia	COVID-19 nucleic acid detection	1, 2, 3	NCT04275245
Fingolimod	2	Parallel	18–80 years	Both	Severe cases of COVID-19 pneumonia	The change of pneumonia severity on X-ray images	NA	NCT04280588
Methylprednisolone	2/3	Parallel	>18 years	Both	RT-PCR confirmed COVID-19 and symptoms for more than 7 days	Lower Murray lung injury score; lower Murray lung injury score	NA	NCT04244591
Bevacizumab	2/3	Single-arm	18–80 years	Both	Confirmed COVID-19 diagnosis	Partial arterial oxygen pressure (PaO2) to fraction of inspiration O2 (FiO2) ratio	1, 2, 3, 6	NCT04275414
Baricitinib	3	Cross-over	18–85 years	Both	Clinical diagnosis of COVID-19 infection and signed an informed consent	The percentage of patients requiring transfer to ICU	1	NCT04320277
Corticosteroid intervention	4	Parallel	>18 years	Both	Patients who are diagnosed with COVID-19	The time of duration of COVID-19 nucleic acid RT-PCR test results of respiratory specimens (such as throat swabs) or blood specimens changes to negative	3, 5, 6	ChiCTR2000030481
Tranilast	4	Parallel	118–85 years	Both	COVID-19 patients with pneumonia and has high IL-1ß level	cure rate	3	ChiCTR2000030002
Tozumab and adamumab	4	Parallel	18–80 years	Both	Severe and critical COVID-19 patients with pneumonia, blood CRP and ESR were more than twice higher than normal or TNF, TNF, and Il-6 were higher than normal	chest computerized tomography, nucleic acid detection of COVID-19, TNF-alpha, IL-6, IL-10	1, 2, 6	ChiCTR2000030580
Cerrokin (recombinant human interferon-alpha 1beta)	4	Parallel	18–110 years	Both	Clinically diagnosed patients with COVID-19	Incidence of side effects	1, 2, 3, 5	ChiCTR2000030480
Anti-bacterial agents								
Carrimycin	4	Parallel	18–75 years	Both	Newly diagnosed COVID-19 patients	Body temperature returns to normal time, pulmonary inflammation resolution time (HRCT), mouthwash (pharyngeal swab) at the end of treatment COVID-19 RNA negative rate	1, 2, 3, 6	ChiCTR2000029867
Anti-viral agents								
Favipiravir tablets	2	Parallel	>18 years	Both	COVID-19 Inpatient with pneumonia	Time to clinical recovery	1, 5	ChiCTR2000029996
Darunavir and cobicistat	3	Parallel	NA	Both	COVID-19 patients with pneumonia	The virological clearance rate of throat swabs, sputum, or lower respiratory tract secretions at day 7	2, 5	NCT04252274
Lopinavir/ritonavir	3	Parallel	>18 months	Both	High-risk close contact with a confirmed COVID-19 case during their symptomatic period	Microbiologic evidence of infection	1, 2, 3	NCT04321174
Triazavirin	3	Parallel	>18 years	Both	RT-PCR and chest imaging (CT) confirmed lung damage	Time to clinical recovery (TTCR)	1, 5, 6	ChiCTR2000030001
Remdesivir	3	Parallel	>18 years	Both	RT-PCR confirmed COVID-19	Time to clinical recovery; time to clinical recovery (TTCR)	2	NCT04252664
Remdesivir	3	Parallel	>18 years	Both	RT-PCR confirmed COVID-19	Time to clinical improvement (TTCI) (Censored at Day 28)	2	NCT04257656
Arbidol tablets	4	Parallel	>18 years	Both	RT-PCR confirmed COVID-19 patients	Virus negative conversion rate in the first week	1, 6	ChiCTR2000029621
Novaferon atomization inhalation and lopinavir/ritonavir tablets	4	Parallel	NA	Both	Light and heavy patients with COVID-19	Time of new coronavirus nucleic acid turning negative	1, 6	ChiCTR2000029496
Chloroquine derivatives								
Chloroquine phosphate	2	Parallel	>18 years	Both	RT-PCR confirmed COVID-19	Viral clearance time	NA	NCT04328493
Hydroxychloroquine	3	Parallel	>18 years	Both	COVID-19 patients with pneumonia	The virological clearance rate of throat swabs, sputum, or lower respiratory tract secretions at day 3, day 5, and day 7. The mortality rate of subjects at week 2	2, 5	NCT04261517
Oral hydroxychloroquine sulfate tablets	4	Parallel	>18 years	Both	COVID-19 patients with pneumonia and onset time <=12 days	Viral nucleic acid test	1, 2, 5, 6	ChiCTR2000029868
Hydroxychloroquine	4	Parallel	30–65 years	Both	COVID-19 patients with pneumonia	The time when the nucleic acid of the novel coronavirus turns negative, T cell recovery time	NA	ChiCTR2000029559
Chloroquine phosphate	4	Parallel	18–70 years	Both	Severe patients with respiratory distress, RR >=30 times/min; in resting state, oxygen saturation <=93%; PaO2/FiO2 <=300 mmHg	Time to clinical recovery	1, 2, 6	ChiCTR2000029988
Chloroquine phosphate	4	Parallel	NA	Both	COVID-19 patients with fever; normal or decreased WBC count or reduced lymphocyte count in the early stages of onset; multiple small patchy shadows and interstitial changes in the extrapulmonary zone	Length of stay, length of severe, oxygenation index during treatment, all-cause mortality in 28 days	1, 2, 6	ChiCTR2000029741
Chloroquine	4	Non-RCT	18–80 years	Both	COVID-19 patients according to WHO interim guidance	Viral negative-transforming time, 30-day cause-specific mortality	1, 2, 5, 6	ChiCTR2000029542
Hydroxychloroquine	4	Parallel	16–99 years	Both	Newly diagnosed COVID-19 with pneumonia	Oxygen index, max respiratory rate, lung radiography, count of lymphocyte, and temperature	1, 2, 3	ChiCTR2000029740
Hydroxychloroquine sulfate	4	Parallel	>18 years	Both	RT-PCR confirmed COVID-19 patients	Time to clinical recovery (TTCR)	1, 2, 5, 6	ChiCTR2000029899
Hydroxychloroquine	4	Parallel	18–75 years	Both	Confirmed COVID-19 patients and not used any antimalarial drugs in the last 3 months	Time to clinical improvement (TTCI)	1, 2, 5, 6	ChiCTR2000029898
Combination therapy								
Hydrochloriqine+azithromycin+tocilizumab	2	Parallel	>18 years	Both	RT-PCR confirmed COVID-19	In-hospital mortality and need for mechanical ventilation in ICU	1, 2, 6	NCT04328285
Ebastine 10 mg bid, interferon-alpha aerosol inhalation 5 million U bid, and lopinavir 200 mg	4	Parallel	NA	Both	RT-PCR detection of nucleic acid positive of COVID-19	Clinical therapeutic course, pathogenic detection, chest CT, laboratory indicators (blood routine, myocardial enzyme spectrum, inflammatory cytokines, etc.)	1, 2, 6	ChiCTR2000030535
Ganovo+ritonavir+/-Interferon	4	Parallel	18–75 years	Both	RT-PCR confirmed COVID-19 patients with pneumonia	Rate of composite adverse outcomes	NA	NCT04291729
Traditional Chinese medicines (TCMs)								
Fuzheng Huayu tablet	2	Parallel	18–65 years	Both	Patients with pulmonary fibrosis after standard treatment of COVID-19	High-resolution computed tomography (HRCT) score; lung function: FVC as a percentage of projected value and diffusing capacity of the lung for CO	NA	NCT04279197
Ba-Bao-Dan	4	Non-RCT	18–90 years	Both	Pathological confirmed COVID-19 patients	Clinical and laboratory indicators; viral load; chest CT; serum cell factor	1, 5	ChiCTR2000029819
Xiyanping injection	4	Parallel	18–70 years	Both	RT-PCR confirmed COVID-19 patients	Clinical recovery time	2, 3	ChiCTR2000030117
Lianhua Qingwen capsules	4	Parallel	>18 years	Both	COVID-19 patients with pneumonia	Clinical symptoms (fever, weakness, cough) recovery rate, and recovery time	1, 2, 3, 6	ChiCTR2000029434
Honeysuckle oral liquid	4	Parallel	NA	Both	COVID-19 patients and symptom onset and randomization were within 7 days	Recovery time, pneumonia PSI score	1, 2, 3, 5, 6, 7	ChiCTR2000029954
Honeysuckle oral liquid	4	Parallel	18–75 years	Both	COVID-19 patients with pneumonia	Recovery time, pneumonia PSI score	1, 2, 3, 6	ChiCTR2000030545
Kesuting syrup	4	Parallel	18–75 years	Both	COVID-19 patients with mild and moderate pneumonia	Cough	1, 3, 5, 6	ChiCTR2000029991
Jinye Baidu granule	4	Parallel	>18 years	Both	Newly diagnosed COVID-19 with pneumonia	Validity observation index	1, 2, 5, 6	ChiCTR2000029755
Shenqi Fuzheng injection	4	Parallel	>18 years	Both	Newly diagnosed COVID-19 with pneumonia	Recovery time	1, 2, 3, 6, 7	ChiCTR2000029780
Kangbingdu granules	4	Parallel	>18 years	Both	Newly diagnosed COVID-19	Disappearance rate of fever symptoms	1, 2, 3, 6, 7	ChiCTR2000029781
Others								
Polyinosinic-polycytidylic acid injection	4	Parallel	NA	Both	COVID-19 patients with pneumonia	Time to clinical recovery	1, 2, 3, 5, 6, 7	ChiCTR2000029776
Sodium aescinate injection	4	Parallel	18–70 years	Both	Diagnosed COVID-19 patients by viral nucleic acid	Chest imaging (CT)	1, 2, 3, 6	ChiCTR2000029742
Inhalate the mycobacterium vaccae	4	Parallel	18–80 years	Both	COVID-19 patients with pneumonia	viral negative-transforming time, 30-day mortality and adverse events	1, 6	ChiCTR2000030016
Alpha-lipoic acid	4	Parallel	35–74 years	Both	COVID-19 patients with severe pneumonia	Sequential organ failure assessment (SOFA)	1, 2, 3, 6	ChiCTR2000029851
Lipoic acid injection	4	Parallel	18–75 years	Both	Mild patients with confirmed COVID-19	Progression rate from mild to critical/severe	1, 2, 3, 6	ChiCTR2000030471

**Table 4:** Summary of COVID-19 therapeutic concepts registered for investigation COVID-19: coronavirus disease 2019; CDC: Centers for Disease Control and Prevention

Start date	Phase	Intervention	Status	Reference	Summary
01-02-2020	Pilot	Recombinant human angiotensin-converting enzyme 2 (rhACE2)	Not yet recruiting	NCT04287686	An open-label, randomized, controlled, pilot study in patients with COVID-19, to obtain preliminary biologic, physiologic, and clinical data in patients with COVID-19 treated with rhACE2 or control patients
01-02-2020	Phase 2/3	Yinhuingwen Decoction	Not yet recruiting	NCT04278963	Randomized, three-arm controlled, single-blind trial to evaluate the efficacy and safety of Yinhu Qingwen decoction in patients hospitalized with mild or common COVID-19
18-02-2020	Phase 2	Thalidomide	Not yet recruiting	NCT04273581	This study will evaluate thalidomide combined with low-dose hormone adjuvant therapy for severe COVID-19 patient effectiveness and safety
20-02-2020	Phase 2	Thalidomide	Not yet recruiting	NCT04273529	This study is the first prospective, multicenter, randomized, double-blind, placebo, parallel controlled clinical study to use immunomodulator-thalidomide in patients with COVID-19
26-02-2020	Not specified	T89	Not yet recruiting	NCT04285190	Open-label, randomized, blank-controlled study focused on investigating the efficacy of T89 on improving oxygen saturation and clinical symptoms in patients with COVID-19
01-03-2020	Phase 2	Nitric oxide gas	Not yet recruiting	NCT04305457	In this study, researchers investigate whether inhaled NO therapy prevents progression in patients with mild to moderate COVID-19 disease
01-03-2020	Phase 3	Remdesivir versus + lopinavir/ritonavir versus lopinavir/ritonavir plus interferon ß-1a	Not yet recruiting	NCT04315948	Multi-centre, adaptive, randomized, open clinical trial of the safety and efficacy of remdesivir versus + lopinavir/ritonavir versus lopinavir/ritonavir plus interferon ß-1a treatments of COVID-19 in hospitalized adults
12-03-2020	Phase 2	Not specified	Not yet recruiting	NCT04280705	Adaptive, randomized, double-blind, placebo-controlled trial to evaluate the safety and efficacy of different novel therapeutic agents in hospitalized adult patients diagnosed with COVID-19
15-03-2020	Phase 2/3	Darunavir/cobicistat plus chloroquine treatment	Not yet recruiting	NCT04304053	The investigators plan to evaluate the efficacy of prophylactic chloroquine treatment to all contacts. The strategy entails decentralized COVID-19 testing and starting antiviral darunavir/cobicistat plus chloroquine treatment immediately in all who are found to be infected
29-03-2020	Phase 2	Inhaled nitric oxide	Not yet recruiting	NCT04312243	Subjects will be randomized either in the observational (control) group or in the inhaled nitric oxide group. All personnel will observe measures on strict precautions in accordance with WHO and the CDC regulations
20-03-2020	Phase 2/3	Yinhu Qingwen	Not yet recruiting	NCT04310865	An adaptive, randomized, double-blind, controlled trial will evaluate the efficacy and safety of Yinhu Qingwen granules in patients hospitalized with severe CoVID-19
01-04-2020	Phase 2	Aviptadil	Not yet recruiting	NCT04311697	Patients will be randomized to intravenous Aviptadil with escalation to nebulized aviptadil vs. nebulized aviptadil with escalation to intravenous aviptadil
01-04-2020	Phase 3	Hydroxychloroquine	Not yet recruiting	NCT04318015	Triple blinded, phase III randomized controlled trial with parallel groups (200 mg of hydroxychloroquine per day vs. placebo) aiming to prove hydroxychloroquine's security and efficacy as prophylaxis treatment for healthcare personnel exposed to COVID-19 patients
01-05-2020	Phase 3	CD24F	Not yet recruiting	NCT04317040	Randomized, placebo-controlled, double-blind, multicenter, Phase III trial to compare Arm A: CD24Fc/Best Available Treatment Arm B: placebo/ Best Available Treatment CD24Fc in hospitalized COVID-19 patients
